# APP antisense oligonucleotides are effective in rescuing mitochondrial phenotypes in human iPSC‐derived trisomy 21 astrocytes

**DOI:** 10.1002/alz.14560

**Published:** 2025-01-29

**Authors:** Srishruthi Thirumalai, Frederick J. Livesey, Rickie Patani, Christy Hung

**Affiliations:** ^1^ Department of Neuroscience City University of Hong Kong Hong Kong Hong Kong; ^2^ Talisman Therapeutics Babraham Research Campus Cambridge UK; ^3^ Human Stem Cells and Neurodegeneration Laboratory The Francis Crick Institute London UK; ^4^ Department of Neuromuscular Diseases Queen Square Institute of Neurology University College London Queen Square London UK; ^5^ UCL Great Ormond Street Institute of Child Health Zayed Centre for Research into Rare Disease in Children London UK

**Keywords:** Alzheimer's disease, antisense oligonucleotides, APP, astrocytes, Down syndrome, mitochondrial function

## Abstract

**INTRODUCTION:**

Antisense oligonucleotides (ASOs) have shown promise in reducing amyloid precursor protein (APP) levels in neurons, but their effects in astrocytes, key contributors to neurodegenerative diseases, remain unclear. This study evaluates the efficacy of APP ASOs in astrocytes derived from an individual with Down syndrome (DS), a population at high risk for Alzheimer's disease (AD).

**METHODS:**

Human induced pluripotent stem cells (hiPSCs) from a healthy individual and an individual with DS were differentiated into astrocytes. Astrocytes were treated with APP ASOs for 10 days, and APP levels were quantified. Mitochondrial morphology and superoxide production in DS astrocytes were analyzed using super‐resolution and confocal microscopy.

**RESULTS:**

APP ASOs significantly reduced APP levels in astrocytes from both control and DS individuals. In DS astrocytes, treatment restored mitochondrial health, increasing mitochondrial number and size while reducing superoxide production.

**DISCUSSION:**

APP ASOs effectively reduce APP levels and improve mitochondrial health in astrocytes, suggesting their potential as a therapeutic approach for DS and DS‐related AD. Further in vivo studies are required to confirm these findings.

**Highlights:**

- APP ASOs reduce APP levels in human iPSC‐derived astrocytes.
- APP ASO treatment rescues mitochondrial phenotypes in trisomy 21 astrocytes.
- This study supports ASOs as a potential therapy for Down syndrome‐related Alzheimer's disease.

## 1 BACKGROUND

Down syndrome (DS), caused by trisomy of human chromosome 21 (trisomy 21), occurs in approximately 1 in 700 live births^1^ and is the most common genetic cause of early‐onset Alzheimer's disease‐dementia (AD‐DS).^2^ Individuals with DS exhibit a wide range of clinical features, including developmental delays, cognitive impairments, and craniofacial abnormalities, alongside higher rates of congenital heart defects, autoimmune disorders, and neurological conditions such as AD.^3^ Globally, around 6 million individuals have DS, with two‐thirds developing clinical dementia by age 65 and nearly all women diagnosed by age 80.

Clinical‐genetic studies have established the amyloid precursor protein (APP) gene on human chromosome 21 as a key driver of early AD onset in individuals with DS.^4^ Overexpression of APP results in increased production and accumulation of amyloid‐beta (Aβ) plaques, a hallmark of AD pathology.

Astrocytes are the most abundant glial cell type in the central nervous system and play crucial roles in neuronal maintenance and support, synaptic activity, neuronal metabolism, and Aβ clearance.^5^, ^6^, ^7^ They respond variably to a wide range of nervous system insults, including traumatic brain injury, spinal cord injury, stroke, inflammation, and neurodegenerative diseases.^8^ Astrocytes are increasingly recognized as fundamental players in AD pathogenesis, with dysfunction in astrocyte calcium signaling leading to network hyperactivity in the early stages of AD.^9^ Various factors triggering reactive astrogliosis have been shown to increase APP expression.^10^ Intraperitoneal injection of lipopolysaccharide (LPS) elevates APP levels in astrocytes in mice and rats.^11^ Similarly, treatment with cytokines such as interleukin 1 (IL1), tumor necrosis factor β (TNFβ), tumor necrosis factor α (TNFα), and interferon γ (IFNγ) increases APP levels in astrocytes both in vitro and in vivo.^10^

**RESEARCH IN CONTEXT**

1. **Systematic review**: We conducted a comprehensive literature review using PubMed and other scientific databases, focusing on the role of antisense oligonucleotides (ASOs) in Alzheimer's disease (AD) and Down syndrome (DS). Previous studies have demonstrated the efficacy of ASOs in reducing amyloid precursor protein (APP) levels in neurons, but there is limited research on their effects on astrocytes, which are crucial in AD pathogenesis.
2. **Interpretation**: Our study demonstrates that APP ASOs significantly reduce APP levels in human induced pluripotent stem cell (hiPSC)—derived astrocytes and rescue mitochondrial dysfunction in DS astrocytes. These findings expand the potential therapeutic applications of ASOs beyond neurons to include astrocytes, suggesting a broader utility in treating AD and related conditions.
3. **Future directions**: Future research should focus on in vivo studies to confirm the efficacy of APP ASOs in astrocytes, investigate long‐term effects, and explore the mechanisms by which ASOs influence astrocyte function and contribute to neuroprotection in AD.

Antisense oligonucleotides (ASOs) have emerged as a promising therapeutic strategy for genetic diseases.^12^ The success of Spinraza, an ASO that modulates the splicing of *SMN2* RNA, provides profound disease‐modifying effects for patients with spinal muscular atrophy, is a prime example of this transformative potential.^13^ While ASOs targeting *APP* mRNA have shown efficacy in reducing APP protein levels in neurons,^14^, ^15^, ^16^ their effects on astrocytes remain unexplored. This study aims to investigate the potential of APP ASOs in astrocytes derived from human induced pluripotent stem cells (hiPSCs) comparing healthy with DS.

## 2 METHODS

### 2.1 Differentiation of hiPSC‐derived astrocytes

hiPSCs were maintained on Geltrex‐coated plates (Life Technologies) in Essential 8 Medium (Life Technologies) and passaged using ethylenediaminetetraactic acid (EDTA). Differentiation of hiPSCs into astrocytes was performed following a previously established protocol.^17^ Details of the cell lines used in this study can be found in Table S1.

Briefly, dissociated hiPSCs were plated on six‐well plates coated with Geltrex and subjected to neural induction by switching to N2B27 medium, a 1:1 mixture of N2 and B27 media, supplemented with 1 µM dorsomorphin and 10 µM SB431542 to inhibit TGFβ signaling during the induction phase. Following neural conversion, the cells entered a propagation phase lasting over 60 days, during which they were maintained in media containing 10 ng/mL fibroblast growth factor 2 (FGF‐2, Peprotech). For terminal differentiation into astrocytes, the cells were treated with bone morphogenetic protein 4 (BMP4, 10 ng/mL, R&D Systems) and leukemia inhibitory factor (LIF, 10 ng/mL, Sigma–Aldrich).

### 2.2 Immunohistochemistry

Samples were fixed in 4% paraformaldehyde for 15 min at room temperature and subsequently blocked in 5% bovine serum albumin (BSA) in phosphate buffered saline (PBS) containing 0.3% Triton X‐100 to permeabilize the cells. Primary antibody staining was performed using chicken anti‐GFAP (Abcam, ab4674) diluted 1:500 in 5% BSA and 0.3% Triton X‐100 in PBS. The samples were incubated with the primary antibody overnight at 4°C.[Fig alz14560-fig-0001], [Fig alz14560-fig-0002]

The next day, samples were washed in PBS and incubated for 1 h at room temperature with Alexa Fluor‐conjugated secondary antibodies (Thermo Fisher), diluted according to the manufacturer's guidelines. Nuclei were counterstained with 4′,6‐diamidino‐2‐phenylindole (DAPI, 100 ng/mL) for 10 min. Finally, samples were mounted with an anti‐fade mounting medium for imaging.

### 2.3 Protein extraction and Western blot analysis

For immunoblotting, whole‐cell lysates were prepared using RIPA buffer (Sigma) supplemented with protease inhibitors (Sigma) and Halt phosphatase inhibitors (ThermoFisher Scientific). Protein concentration was determined using the Precision Red Advanced Protein Assay buffer (Cytoskeleton, Inc.). Equal amounts of protein were loaded onto a 4%–12% sodium dodecyl sulfate‐polyacrylamide gel electrophoresis (SDS‐PAGE) gel, separated by electrophoresis, and transferred onto polyvinylidene fluoride (PVDF) membranes.

Membranes were blocked with LI‐COR blocking buffer for 1 h at room temperature and then incubated overnight at 4°C with primary antibodies, including mouse anti‐APP (Cat # 802801, BioLegend), and anti‐β‐actin (Cat #A2228, Sigma), diluted according to the manufacturer's instructions. After washing, membranes were incubated with species‐specific near‐infrared fluorescent secondary antibodies (IRDye, LI‐COR) for 1 h at room temperature. Proteins were visualized using the LI‐COR Odyssey imaging system.

### 2.4 Mitochondrial phenotypes analysis

Mitochondrial morphology in astrocytes was assessed using MitoTracker Red FM Dye staining. The dye was reconstituted in dimethyl sulfoxide (DMSO) to create a 1 mM stock solution and diluted to a final concentration of 1 µM in neurobasal media. Astrocytes were incubated with 250 µL of the staining solution for 30 min at 37°C. Live‐cell imaging was conducted using an iSIM microscope equipped with a 150× oil objective and deconvolution capability. Z‐stack images were captured at a 0.25 µm step size.

The images were analyzed using Imaris software, where 3D renderings of mitochondria were generated. Regions of interest were identified, and thresholding was applied following background subtraction. Mitochondrial parameters, including total number, surface area, intensity, and volume, were quantified using the Statistics function in Imaris.

Mitochondrial superoxide production was assessed using MitoSOX Mitochondrial Superoxide Indicators according to the manufacturer's protocol. The dye was reconstituted in 13 µL of anhydrous DMSO to produce a 5 mM stock solution and diluted to a final concentration of 500 nM in neurobasal media. Astrocytes were incubated with 250 µL of the staining solution for 30 min at 37°C. Live‐cell imaging was performed using a Zeiss SP8 confocal microscope equipped with a CO_2_ environment chamber. Fluorescent intensity, indicative of mitochondrial superoxide levels, was quantified using ImageJ software.

### 2.5 ASOs

DS astrocytes were treated with either a 20‐mer gapmer control ASO or an APP ASO designed to hybridize with exon 5 of the *APP* mRNA. Both ASOs (Integrated DNA Technologies, IDT) were synthesized with a phosphorothioate (PS) backbone for stability and featured a central gap of ten 2′‐deoxynucleotides flanked by five 2′‐O‐methoxyethyl (MOE)‐modified nucleotides on each side to enhance nuclease resistance. To further reduce degradation, all cytidine residues in the central gap region were substituted with 5‐methyl‐deoxycytidine. The sequence of the APP ASO was 5′‐TGTCACTTTCTTCAGCCAGT‐3′.

ASOs were prepared as a 50 µM stock solution by dissolving lyophilized oligonucleotides in nuclease‐free water. The APP ASO was added to DS astrocytes at a final concentration of 1.75 µM, with the medium containing the ASO replaced every 3 days over a 10‐day treatment period.

To serve as a negative control, a scrambled sequence ASO (control ASO) with the same chemical modifications as the APP ASO was used. This control allowed us to distinguish sequence‐specific silencing effects from potential non‐specific off‐target effects in DS astrocytes.

### 2.6 Statistical analysis

Unless otherwise specified, statistical analyses were performed using GraphPad Prism (Version 10). A Student's *t*‐test was used to compare differences between the two groups, with a significance threshold set at *p* < 0.05. Data are presented as mean ± standard error of the mean (SEM).

## 3 RESULTS

**FIGURE 1 alz14560-fig-0001:**
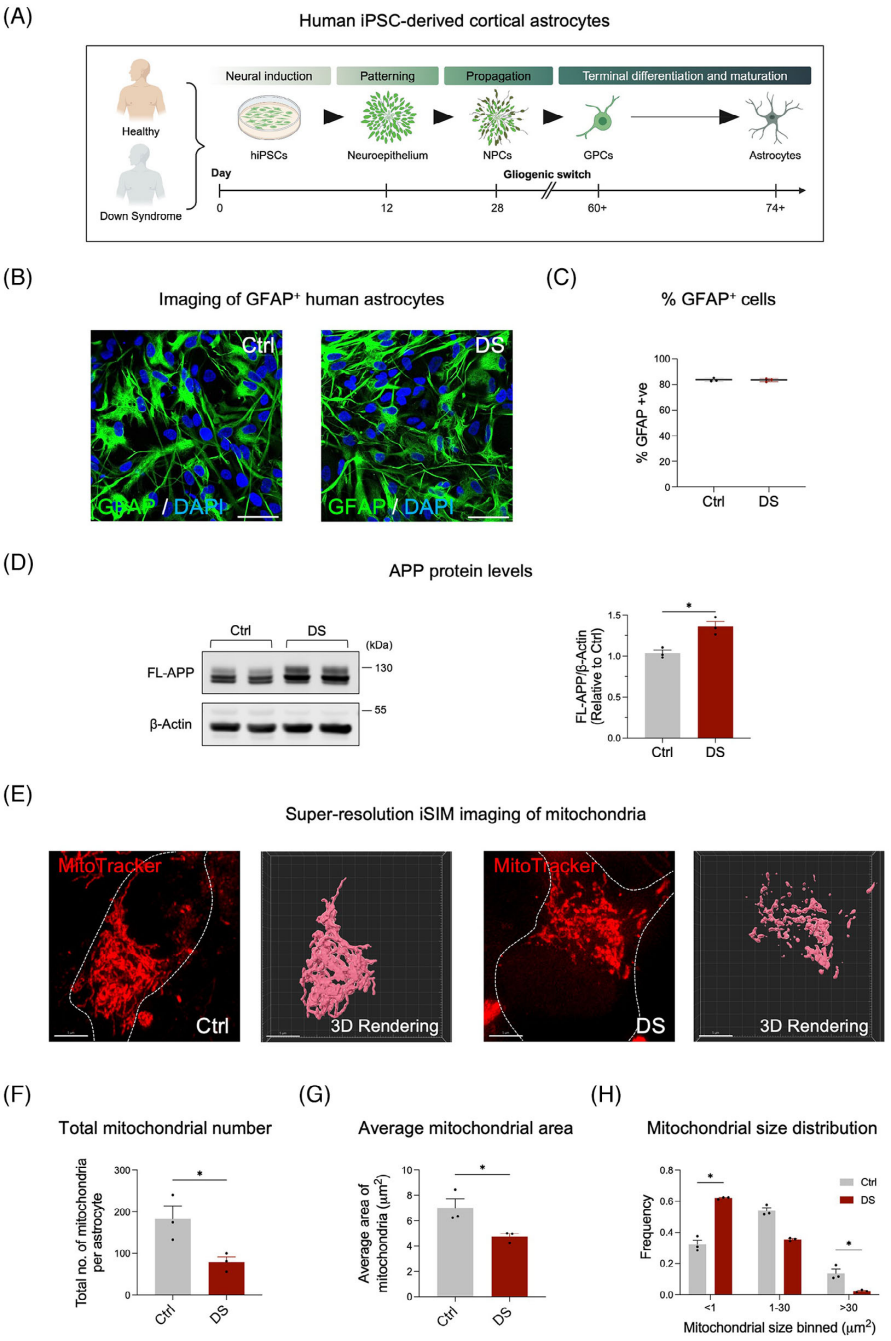
(A) Schematic illustrating the directed differentiation strategy used to specify cortical astrocytes from hiPSCs. Experiments were performed in three technical replicates (*n* = 3) for each condition, with independent astrocyte differentiations serving as biological replicates. Representative immunofluorescence images (B) and quantification (C) of GFAP‐positive cells (green) in control and DS astrocytes. Scale bar = 50 µm. Quantification includes three technical replicates per condition (*n* = 3), derived from five randomly selected fields of view per replicate, imaging over 50 cells per field. Data are represented as mean ± SEM. Significance was calculated using an unpaired Student's *t*‐test. (D) Representative Western blots showing full‐length APP and β‐actin (loading control) in control and DS astrocytes (*n* = 3 independent experiments). Data are represented as mean ± SEM, with significance calculated using an unpaired Student's *t*‐test. *p < 0.05. (E) Representative images and 3D renderings of mitochondria labeled with MitoTracker Red in control and DS astrocytes. Scale bar = 5 µm. Quantification of mitochondrial metrics: total number (F), average area (G), and size distribution (H) in control and DS astrocytes. Data represent three technical replicates per condition (*n* = 3), with each replicate derived from three randomly selected fields of view, imaging at least 10 cells per field. Data are represented as mean ± SEM, with significance calculated using an unpaired Student's *t*‐test. *p < 0.05. APP, amyloid precursor protein; DS, Down syndrome; GFAP, glial fibrillary acidic protein; hiPSCs, human induced pluripotent stem cells; SEM, standard error of the mean.

We sought to determine whether APP ASOs would effectively reduce APP levels in hiPSC‐derived astrocytes. We first used our previously published directed differentiation paradigm to generate highly enriched populations of hiPSC‐derived astrocytes from a healthy individual and an individual with DS harboring three copies of the APP gene (Figure 1A).^17^ To assess the enrichment of astrocytes following the differentiation of glial precursors, we performed quantitative immunocytochemistry (qICC) for glial fibrillary acidic protein (GFAP), one of the principal intermediate filaments in mature astrocytes. Both control and DS cells expressed high levels of GFAP, confirming their robust differentiation into astrocytes (Figure 1B,C). Consistent with our previous findings in hiPSC‐derived cortical neurons,^15^ we observed that increased APP gene dosage elevates full‐length APP protein levels in hiPSC‐derived astrocytes (Figure 1D).

Mitochondria are critical for cellular energy production, reactive oxygen species (ROS) regulation, calcium homeostasis, and apoptotic signaling—all processes essential for astrocytic function and neuronal support. Alterations in APP processing have been associated with mitochondrial dysfunction in human fetal DS cortical astrocytes.^18^ Using super‐resolution instant structured illumination microscopy (iSIM) (Figure 1E), we analyzed mitochondrial morphology in control and DS astrocytes. A significant reduction was observed in the total number of mitochondria (Figure 1F) and the average mitochondrial area (Figure 1G) in DS astrocytes compared to controls. Furthermore, the proportion of elongated mitochondria (with a size exceeding 30 µm^2^) was markedly decreased in DS astrocytes (Figure 1H).

**FIGURE 2 alz14560-fig-0002:**
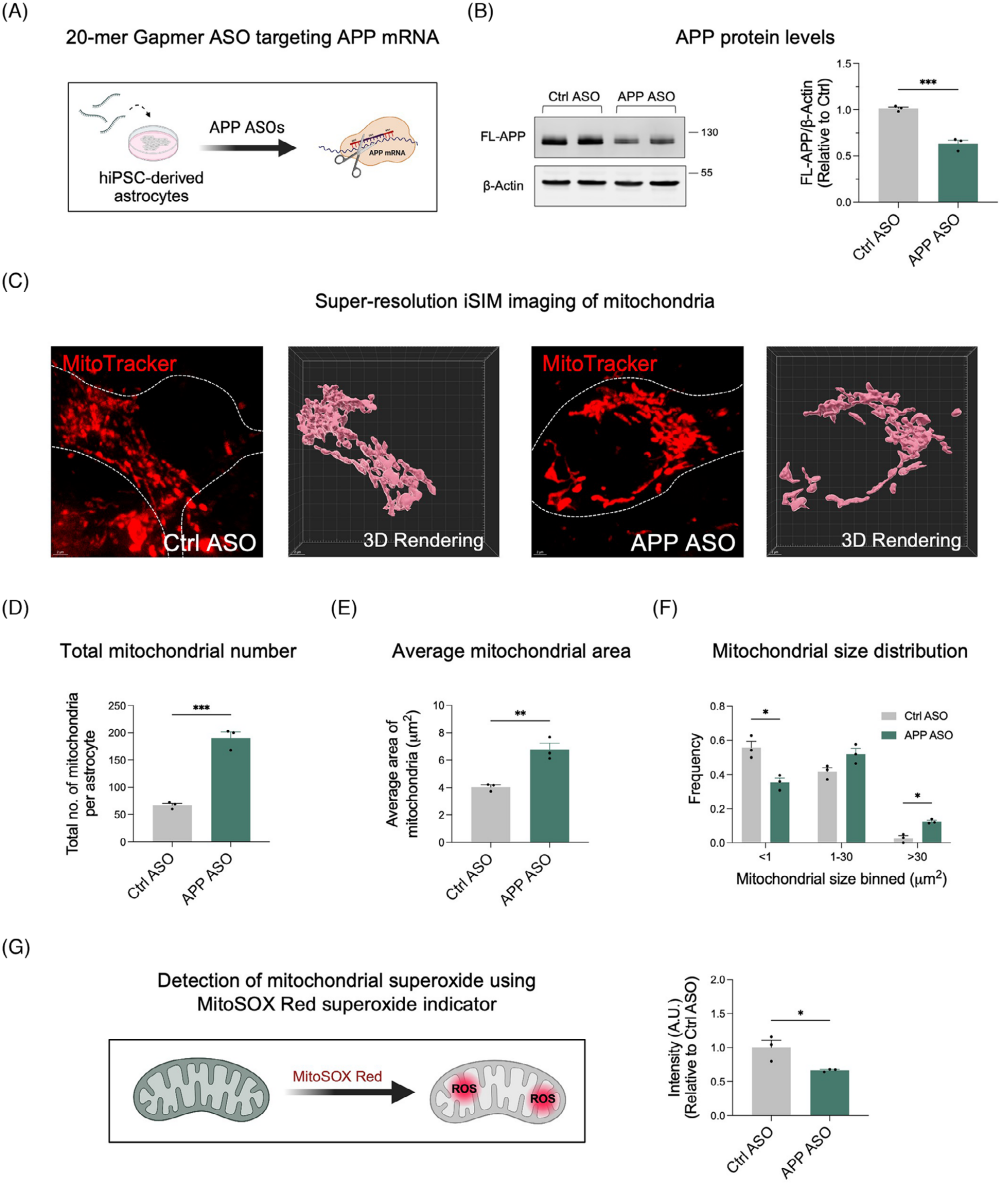
(A) Schematic of APP antisense oligonucleotide (ASO) binding regions and experimental design. (B) Representative Western blots of full‐length APP and β‐actin in astrocytes treated with either control ASOs or APP ASOs (*n* = 3 independent experiments). Data are represented as mean ± SEM, with significance calculated using an unpaired Student's *t*‐test. ***p < 0.001. (C) Representative images and 3D renderings of mitochondria labeled with MitoTracker Red in DS astrocytes treated with control ASOs or APP ASOs. Scale bar = 2 µm. Quantification of mitochondrial metrics: total number (D), average area (E), and size distribution (F) in DS astrocytes treated with control ASOs or APP ASOs. Data points represent three technical replicates per condition (*n* = 3), with each replicate derived from three randomly selected fields of view, imaging at least 10 cells per field. Data are represented as mean ± SEM, with significance calculated using an unpaired Student's *t*‐test. *p < 0.05, **p < 0.01, ***p < 0.001. (G) Quantification of mitochondrial superoxide production in DS astrocytes treated with control ASOs or APP ASOs. Data points represent three technical replicates per condition (*n* = 3), with each replicate derived from three randomly selected fields of view, imaging at least 50 cells per field. Data are represented as mean ± SEM, with significance calculated using an unpaired Student's *t*‐test. *p < 0.05. APP, amyloid precursor protein; ASOs, antisense oligonucleotides; DS, Down syndrome; SEM, standard error of the mean

We previously employed a 20‐mer gapmer APP ASO targeting Exon 5 of the *APP* mRNA and demonstrated that APP ASOs are effective in reducing APP levels in hiPSC‐derived cortical neurons.^14^, ^15^ This reduction brought APP levels from those expected with three copies of the gene down to levels expected from two copies (approximately a 33% reduction). To determine if APP ASOs are similarly effective in hiPSC‐derived astrocytes, we treated these astrocytes with the same concentration of APP gapmer ASO used in our hiPSC‐derived cortical neuron experiments for 10 days (Figure 2A). We observed a significant decrease in APP levels in hiPSC‐derived astrocytes treated with APP ASOs compared to those treated with control ASOs (Figure 2B). These findings confirm that APP ASOs effectively reduce APP levels in hiPSC‐derived astrocytes.

We hypothesized that restoring physiological APP protein levels would rescue mitochondrial dysfunction in DS astrocytes. To test this, we treated DS astrocytes with APP ASOs for 10 days and analyzed mitochondrial morphology using super‐resolution iSIM (Figure 2C). APP ASO treatment significantly increased the total number of mitochondria (Figure 2D), the average mitochondrial area, and the proportion of elongated mitochondria (size > 30 µm^2^) (Figure 2E,F), suggesting improved mitochondrial health.

To further investigate the functional impact of APP ASO treatment, we measured mitochondrial superoxide production using MitoSOX mitochondrial superoxide indicators and live‐cell imaging. Mitochondria are an important source of ROS, which play dual roles in cellular signaling and regulation but contribute to cellular damage under pathological conditions.^19^, ^20^ MitoSOX is a fluorogenic dye that specifically targets mitochondria in live cells, producing bright red fluorescence upon oxidation by superoxide.^21^ DS astrocytes treated with APP ASOs exhibited a significant reduction in fluorescent intensity compared to those treated with control ASOs, indicating decreased mitochondrial ROS production (Figure 2G). These findings suggest that APP ASO treatment not only improves mitochondrial morphology but also mitigates oxidative stress, further supporting its therapeutic potential.

## 4 DISCUSSION

This study demonstrates that APP ASOs effectively reduce APP levels in astrocytes, expanding the potential therapeutic application of ASOs beyond neurons to include glial cells. Given the crucial role of astrocytes in AD pathogenesis, these findings suggest that ASOs may be a versatile tool in treating AD by targeting multiple cell types within the brain.

The observed improvement in mitochondrial function in DS astrocytes following ASO treatment highlights a potential mechanism by which APP reduction can ameliorate cellular dysfunction in AD. This supports the hypothesis that APP overexpression contributes to mitochondrial impairment and suggests that targeting APP in astrocytes may provide neuroprotection.

However, the long‐term effects of ASO treatment on astrocytes and their implications for AD progression require further investigation.^16^ Future research should focus on in vivo studies to confirm the efficacy of APP ASOs in astrocytes, investigate the long‐term safety and effectiveness of ASOs across various brain cell types, and explore the molecular mechanisms by which ASOs modulate astrocyte function.

## CONFLICT OF INTEREST STATEMENT

The authors declare no conflicts of interest. Author disclosures are available in the Supporting information.

## Supporting information



Supporting Information

Supporting Information
