# Supplementary material for: APP antisense oligonucleotides are effective in rescuing mitochondrial phenotypes in human iPSC‐derived trisomy 21 astrocytes
Source: Alzheimers Dement. 2025 Jan 29;21(1):e14560. doi: 10.1002/alz.14560 (PMC11775556; doi:10.1002/alz.14560)
Supplement: Supplementary file 1 — Supporting Information [file ALZ-21-e14560-s001.pdf]

Supplementary information

Control/patient	Cell line name	Source	Age at sampling	Sex of cell	Cell type
Control	UCSD224i-NDC1-2	University of California - San Diego	86Y	M	Fibroblast of skin
Down syndrome	DS1-iPS4	Children's Hospital Boston; Boston; USA	1Y	M	Fibroblast of skin

Supplementary table 1. Details of the cell lines used in this study.
